# Paraneoplastic Minimal Change Disease Signaling Post-Transplant AML Relapse: Two Cases and a Literature Review

**DOI:** 10.3390/curroncol33070382

**Published:** 2026-06-24

**Authors:** Kainat Saleem, Sanjana Kamat, Nigar A. Khurram, Bassem S. Hendawy, Sawa Ito, Pooja Amarapurkar

**Affiliations:** 1 Division of Malignant Hematology and Medical Oncology, Department of Medicine, University of Pittsburgh Medical Center, Pittsburgh, PA 15232, USA; 2 Division of Internal Medicine, Conemaugh Memorial Medical Center, Johnstown, PA 15905, USA; 3 Division of Renal and Transplantation Pathology, Department of Pathology, University of Pittsburgh School of Medicine, Pittsburgh, PA 15213, USA; 4 Division of Renal and Electrolyte Medicine, Department of Medicine, University of Pittsburgh School of Medicine, Pittsburgh, PA 15213, USA

**Keywords:** acute myeloid leukemia, minimal change disease, nephrotic syndrome, hematopoietic stem cell transplantation, paraneoplastic glomerulonephritis, AML relapse, graft-versus-host disease, podocytopathy

## Abstract

Membranous nephropathy (MN) and minimal change disease (MCD) are the most common causes of nephrotic syndrome following hematopoietic stem cell transplantation (HSCT), a complication conventionally attributed to chronic graft-versus-host disease (GVHD). Paraneoplastic MCD is well described in lymphoid malignancies but is rarely reported in myeloid neoplasms. We report two cases of biopsy-confirmed MCD presenting as the initial manifestation of acute myeloid leukemia (AML) relapse following allogeneic HSCT. Both patients were White men in their sixties with relapsed/refractory AML who developed nephrotic-range proteinuria and acute kidney injury after matched unrelated donor HSCT without histologic evidence of GVHD. Renal biopsies confirmed MCD in both cases. Corticosteroid therapy was ineffective in halting renal deterioration; renal function improved only after initiation of leukemia-directed therapy, with one patient achieving dialysis independence. These cases highlight a rare paraneoplastic presentation of AML relapse. Nephrotic syndrome due to MCD may signal post-HSCT leukemia recurrence, and evaluation for AML relapse warrants consideration in steroid-refractory cases or those without concurrent GVHD. In such cases, control of the underlying malignancy, rather than escalation of immunosuppression, may be central to renal recovery.

## Simple Summary

Stem cell transplantation is a treatment for blood cancers such as acute myeloid leukemia. Some patients develop a kidney condition called nephrotic syndrome after transplant, where kidneys leak large amounts of protein into the urine, causing swelling and low blood protein levels. This condition is usually linked to a transplant complication called graft-versus-host disease (GVHD), where donor immune cells attack the patient’s tissues. We describe two patients who developed nephrotic syndrome from minimal change disease after transplant, not from GVHD, but as a manifestation of leukemia relapse. In both cases, kidney dysfunction remained refractory to standard steroid therapy and improved only after leukemia-directed treatment was initiated. These cases suggest that in post-transplant patients with steroid-resistant nephrotic syndrome and absence of active GVHD, clinicians should promptly evaluate for unrecognized leukemia relapse so that leukemia-directed therapy is not inappropriately delayed.

## 1. Introduction

Allogeneic hematopoietic stem cell transplantation (HSCT) remains a potentially curative approach for high-risk hematologic malignancies such as myelodysplastic syndrome (MDS) and acute myeloid and lymphoid leukemias (AML, ALL). Despite ongoing advances in transplant techniques, conditioning regimens and supportive care, renal injury remains a common and clinically consequential complication of the procedure. Acute kidney injury (AKI) develops in approximately 55–65% of patients undergoing HSCT, and 18–66% of long-term survivors eventually develop chronic kidney disease (CKD), a range that reflects variability in study definitions and follow-up duration across the literature [1,2,3,4].

The causes of post-HSCT renal injury are numerous and may involve the vascular, glomerular, or tubulointerstitial compartments, often in an overlapping manner [4,5]. Contributing factors include pre-existing comorbidities such as hypertension, diabetes, as well as hemodynamic perturbations during the transplant course, infections, nephrotoxic effects of conditioning and immunosuppressive agents, radiation-related injury, and transplant-specific complications including thrombotic microangiopathy, hepatic veno-occlusive disease, and graft-versus-host disease (GVHD). Importantly, the dominant pathogenic mechanisms shift across distinct temporal phases of the post-transplant course, giving rise to characteristic clinical phenotypes at each stage [4,6].

In the late post-engraftment period, generally beyond six months after transplantation, nephrotic syndrome (NS) emerges as the predominant form of renal injury. Defined by urinary protein excretion exceeding 3.5 g per 24 h in association with hypoalbuminemia, hyperlipidemia, and peripheral edema, NS in this setting most commonly reflects immune-mediated glomerular injury. Membranous nephropathy (MN) and minimal change disease (MCD) are the most frequently identified histologic subtypes, with MN predominating and MCD accounting for approximately 20% of cases [6,7,8]. Most post-HSCT NS cases arise in temporal association with chronic GVHD or tapering of immunosuppression, consistent with an alloreactive immune mechanism directed at glomerular structures.

NS does, however, occasionally develop in the absence of overt GVHD, and the mechanism in such cases is not well established. Outside the HSCT setting, both MN and MCD are recognized paraneoplastic phenomena with well-documented associations with solid tumors and lymphoid malignancies; their occurrence in myeloid disease is considerably less common. Whether underlying malignancy relapse can drive post-HSCT NS through a paraneoplastic mechanism has not been previously described.

Here, we report two patients who developed biopsy-proven MCD after allogeneic HSCT, each in close temporal proximity to rising AML disease burden, and without histologic evidence of renal GVHD or leukemic infiltration. In both cases, renal function recovered only after initiation of leukemia-directed therapy. These cases suggest that paraneoplastic MCD may serve as an early harbinger of post-HSCT AML relapse and highlight the importance of evaluating for underlying disease recurrence in this setting.

## 2. Detailed Case Description

### 2.1. Case 1

The patient was a White man in his early 60s, with a history of relapsed/refractory AML with MDS-related changes (cytogenetics: t(2;12) and del(5q); molecular: ASXL1 and BCOR mutations). He underwent allogeneic peripheral blood HSCT from a female, HLA-matched unrelated donor (MUD) approximately 18 months after initial diagnosis, receiving reduced-intensity conditioning with fludarabine, busulfan and anti-thymocyte globulin (ATG). Pre-HSCT bone marrow biopsy demonstrated complete remission with no measurable residual disease (MRD) by multiparametric flow cytometry, a response that was maintained at the one-year post-HSCT evaluation. His post-HSCT course was complicated by mild to moderate chronic GVHD involving the skin, oral mucosa and lungs, treated with topical steroids and FAM therapy (fluticasone, azithromycin, montelukast), respectively. He did not require systemic steroids for GVHD management.

Two years post-HSCT, he presented to his primary care physician with two-week history of progressive bilateral lower extremity edema, abdominal distension, and dyspnea on exertion. Vitals showed blood pressure 128/88 mmHg, pulse 114 bpm, and oxygen saturation 93% on room air. He weighed 95 kg compared to a baseline of 86 kg. He was admitted for further evaluation. Pertinent lab findings (Table 1) included AKI with serum creatinine 3.14 mg/dL (baseline 0.8–1 mg/dL), BUN 32 mg/dL (baseline 5–6 mg/dL), and eGFR 21.6 mL/min/1.73 m^2^. Urinalysis showed large blood and spot urine protein > 1000 mg/dL. Urine sediment microscopy showed 5–7 monomorphic RBCs per high-power-field (HPF), 0–2 WBCs per HPF, fine granular casts, and no cellular casts. Peripheral blood smear was negative for schistocytes, arguing against systemic thrombotic microangiopathy. Serologic testing was notable for a weakly positive ANA (titer 1:80, speckled pattern) and a positive anti-MPO antibody (>8.0 AI; reference < 0.1 AI); both were considered non-specific findings in this context. Complement C3 was normal and C4 mildly low at 12 mg/dL (reference range 16–38 mg/dL). HIV, hepatitis B and C serologies and anti-dsDNA, anti-chromatin, anti-GBM, anti-PLA2R, and anti-PR3 antibodies were all negative. Cryoglobulins were negative and renal ultrasonography was unremarkable.

**Figure 1:**
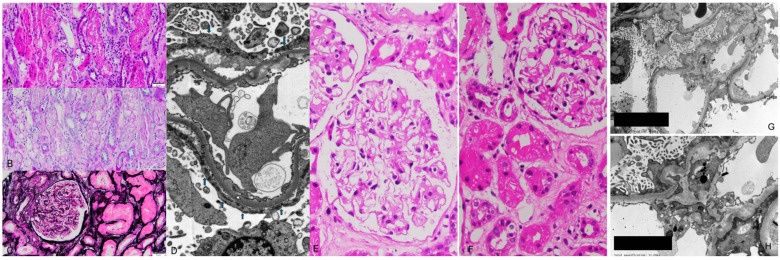
Representative renal biopsy sections. Patient 1—(**A**): Renal tubules showing acute tubular injury in the form of tubular cell ballooning, and isometric-type intracytoplasmic vacuolization (H&E, 200×); (**B**): Renal tubules showing acute tubular injury in the form of tubular cell ballooning, cytoplasmic vacuolization and patchy loss of brush border (PAS, 200×); (**C**): Glomerulus with normal morphology. Tubules show abnormal contour indicating tubular injury (JMS, 200×); (**D**): Electron microscopy (ultrastructural examination, 9300×): glomerular basement membrane (GBM, white star) with normal morphology and thickness (~340 nm) and no evidence of immune-complex deposits. Podocytes foot processes (blue arrows) with semidiffuse fusion/effacement and focal detachment from the underlying GBM. Patient 2—(**E**,**F**): Glomeruli are normocellular with patent capillary loops, delicate non-thickened basement membranes, and no mesangial expansion, segmental sclerosis, or endocapillary hypercellularity. Tubules, interstitium, and vessels are unremarkable, without significant tubular atrophy or interstitial fibrosis (H&E, 200×); (**G**,**H**): Transmission electron microscopy demonstrates diffuse effacement of podocyte foot processes overlying a glomerular basement membrane of normal thickness and texture, with associated microvillous transformation of the podocyte surface. No electron-dense immune-type deposits are identified in the mesangial, subendothelial, or subepithelial compartments (ultrastructural examination, G 4800×; H, 11,000×).

A renal biopsy was performed, and empiric pulse methylprednisolone (1 g IV daily for 3 days) was initiated while awaiting the results. On light microscopy, there was no endocapillary proliferation, fibrin thrombi, necrotizing lesions, or crescents. Ultrastructural examination demonstrated a glomerular basement membrane of normal thickness, with enlarged podocytes showing focal microvillous transformation and diffuse, severe foot process effacement involving nearly the entire capillary surface. No electron-dense or immune complex-type deposits, tubuloreticular inclusions, fibrin tactoids, or fibrillary deposits were identified. The tubulointerstitial compartment showed only mild, non-specific acute tubular injury (Figure 1A–D). The biopsy showed no evidence of vasculitis or thrombotic microangiopathy. The overall findings were consistent with a podocytopathy, specifically minimal change disease. Despite corticosteroid therapy, the patient’s renal function deteriorated with serum creatinine peaking at 5.7 mg/dL, necessitating initiation of intermittent hemodialysis.

Concurrently, the patient developed progressive pancytopenia and circulating blasts. Repeat bone marrow biopsy showed a hypocellular marrow (20%) with 20–25% CD34+ blasts, and re-emergence of the original leukemic clone with t(2;12), del(5q) and the ASXL1 mutation, findings consistent with AML relapse. Treatment with azacitidine, venetoclax and gemtuzumab ozogamicin was initiated. A follow-up bone marrow biopsy at four weeks demonstrated a morphological leukemia free state with only 0.02% leukemic blasts detected by multiparametric flow cytometry, consistent with MRD-positive remission. Renal recovery paralleled the leukemia response, with normalization of urine output, improvement of serum creatinine to baseline, and resolution of proteinuria. Although a second HSCT was planned to follow three cycles of therapy, the patient succumbed to infectious complications during a prolonged period of neutropenia.

### 2.2. Case 2

The patient was a White man in late 60s with a history of relapsed/refractory AML (cytogenetics: t(1;14) and monosomy 7; molecular: IDH2 and EZH2 mutations). He underwent allogeneic peripheral blood HSCT from a male MUD with the same reduced-intensity conditioning regimen of fludarabine, busulfan and ATG. Pre-HSCT bone marrow biopsy demonstrated a morphological leukemia-free state with no MRD by flow cytometry. His post-HSCT course was complicated by grade 1 acute cutaneous GVHD treated with topical steroids and presumed lower GI acute GVHD treated with a short course of oral prednisone. His AML relapsed on day 100 post-HSCT, with bone marrow demonstrating 2.2–2.5% blasts, MRD positivity by flow cytometry, and del(7q) by FISH. Salvage therapy was initiated with azacitidine, followed by an investigational regimen combining interferon gamma with donor lymphocyte infusion. However, his leukemia progressed through both treatments.

At a subsequent oncology visit to discuss further treatment options, he was incidentally noted to have new-onset bilateral lower extremity edema and decreased urine output. Laboratory evaluation (Table 1) revealed AKI with serum creatinine 1.8 mg/dL (baseline 0.7–0.9 mg/dL), BUN 43 mg/dL, eGFR 40 mL/min/1.73 m^2^, hypoalbuminemia, and spot urine protein > 1000 mg/dL. A 24-h urine collection showed marked proteinuria at 16.82 g/day. Serological workup demonstrated low-titer ANA positivity (1:80; speckled) considered to be a non-specific finding in this context. Complement levels, ANCA and hepatitis B testing were normal.

A renal biopsy was performed, and empiric prednisone (60 mg daily) was initiated. Decitabine and venetoclax were initiated concurrently for the refractory AML. On light microscopy, there were no hyaline thrombi, hyalinosis lesions, glomerular basement membrane spikes, or double contours, and immunofluorescence showed no definite immune complex deposits. Ultrastructural examination demonstrated a glomerular basement membrane of normal thickness (approximately 400 nm) with 50–60% effacement of the podocyte foot processes and associated microvillous transformation. The tubulointerstitial compartment showed mild to focally moderate acute tubular injury (Figure 1E–H). The overall findings were consistent with minimal change disease.

Within three days of initiation of leukemia-directed therapy, spot urine protein fell to 261 mg/dL, indicating rapid reduction in glomerular protein leak. Serum creatinine normalized within one month, coinciding with completion of the first treatment cycle. Although the patient had an initial hematologic response, the leukemia ultimately proved refractory and he transitioned to hospice care.

## 3. Discussion

Post-HSCT nephrotic syndrome is most often attributed to chronic GVHD or immunosuppression withdrawal; however, the cases presented here illustrate a clinically distinct and under-recognized etiology: paraneoplastic glomerular injury associated with AML relapse that may warrant a different diagnostic and therapeutic approach. While rare cases of post-HSCT MCD in the setting of AML relapse have been reported previously, our cases are notable for demonstrating a close temporal association between renal recovery and improvement in leukemic disease burden following leukemia-directed therapy.

### 3.1. Post-HSCT Nephrotic Syndrome: Established Context and Diagnostic Gaps

The reported incidence of post-HSCT nephrotic syndrome ranges from 0.8% to 6%, with a median onset of approximately 20 months following transplantation [5,9,10,11]. Histologically, MN accounts for the majority of cases, with MCD diagnosed in an estimated 20% of NS patients [7,11]. Both entities are considered immune-mediated and conventionally linked to chronic GVHD, though rare cases arising in the absence of GVHD have been reported, and their pathophysiologic basis remains incompletely understood [11,12,13,14]. Anti-FAT1 and anti-PLA2R serum antibody and immune complex detection in the glomerular basement membrane by IHC/IF may be useful diagnostic tools for Post-HSCT MN [15,16].

In both of our patients, the initial presentation of nephrotic syndrome raised GVHD as the leading diagnostic consideration, given their post-HSCT status and histories of prior, though well-controlled, GVHD. Several features, however, argued against a GVHD mechanism. First, renal biopsies in both cases demonstrated minimal change disease. Although MCD has been reported as a rare manifestation of renal GVHD, the predominant histologic pattern is membranous nephropathy, often accompanied by subendothelial deposits, tubuloreticular inclusions, and tubular basement membrane deposits, features that were absent in both of our patients [17]. Other recognized presentations of renal GVHD, such as tubulointerstitial nephritis, were likewise not observed. Second, neither patient had evidence of concurrent GVHD activity in other organ systems at the time of nephrotic syndrome onset. Third, and most importantly, high-dose corticosteroid therapy, the cornerstone of GVHD management, failed to arrest deterioration of renal function in either case. Renal recovery followed initiation of leukemia-directed therapy and tracked closely with reduction in leukemic disease burden. This clinical pattern, in which steroid-refractory disease responded promptly to anti-leukemic treatment, points toward a paraneoplastic rather than GVHD-mediated mechanism.

### 3.2. Paraneoplastic Glomerular Disease in Myeloid Malignancies

Paraneoplastic glomerular diseases are well characterized in the context of solid tumors, particularly carcinomas of the lung, ovary, and pancreas, and in lymphoid malignancies including Hodgkin’s lymphoma, non-Hodgkin’s lymphoma, and chronic lymphocytic leukemia [18]. The proposed mechanisms in these conditions involve immune cross-reactivity between tumor-associated antigens and glomerular components, as well as dysregulated production of soluble mediators such as IL-13 and VEGF, although the predominant pathophysiology varies by malignancy.

In contrast, paraneoplastic nephropathy arising in the context of myeloid neoplasms is distinctly uncommon, and its true incidence remains unknown. Among a cohort of 114 patients with MDS, nephrotic syndrome was reported in only 2.4% [19]. In this disease group, dysregulated cytokine production, particularly tumor necrosis factor-α (TNF-α), and direct glomerular deposition of leukemia-associated immune complexes derived from MDS-mediated autoimmunity have been proposed as the main drivers [20]. Glomerular injury in other myeloid conditions arises through different mechanisms. In chronic myelomonocytic leukemia (CMML), urinary lysozyme released from neoplastic monocytes can cause proximal tubular injury and nephrotic-range non-albumin proteinuria, producing a clinically misleading “pseudo-nephrotic syndrome.” In chronic myelogenous leukemia (CML), dysregulated tyrosine kinase signaling mediated by the BCR-ABL1 fusion protein has been implicated in glomerular pathology [18]. In Philadelphia chromosome-negative myeloproliferative neoplasms such as polycythemia vera, essential thrombocythemia, and primary myelofibrosis, dysregulated platelet activation and microvascular endothelial injury are thought to contribute to paraneoplastic focal segmental glomerulosclerosis (FSGS) and mesangial proliferative glomerulonephritis [20,21]. The mechanistic diversity reflects the underlying biologic heterogeneity of myeloid neoplasms.

### 3.3. AML-Associated Nephrotic Syndrome: Literature Review

Paraneoplastic nephrotic syndrome attributable to AML is exceedingly rare, and the published literature consists of scattered case reports, most from the 1980s and 1990s, with only a few described in the past decade and very few reported in the post-HSCT setting (Table 2). Reported histologic subtypes include MCD, MN, and FSGS, though in several cases, biopsy was not performed due to thrombocytopenia, and the diagnosis was inferred from clinical and demographic features alone, an important limitation when interpreting the reported histologic distribution. The timing of NS diagnosis relative to AML varied considerably across reports: some cases preceded the AML diagnosis, others presented concurrently, and others emerged during or after cytotoxic chemotherapy, making it difficult to draw uniform conclusions about causality.

The case reported by Dosa et al. is particularly instructive [22]. This patient developed nephrotic syndrome during active AML, achieved renal remission when the leukemia was treated effectively, and then relapsed renally in close temporal proximity to leukemic relapse, a clinical course closely paralleling that of both patients reported here. Notably, this report identified leukemia-associated antigens and antibodies directed against leukemic cell membrane antigens within glomerular immune deposits, providing direct histochemical evidence for immune cross-reactivity between the malignant clone and glomerular structures. This study remains among the very few published reports offering mechanistic insight into AML-associated nephrotic syndrome, and the underlying pathophysiology warrants further investigation.

### 3.4. Management Considerations

**Figure 2:**
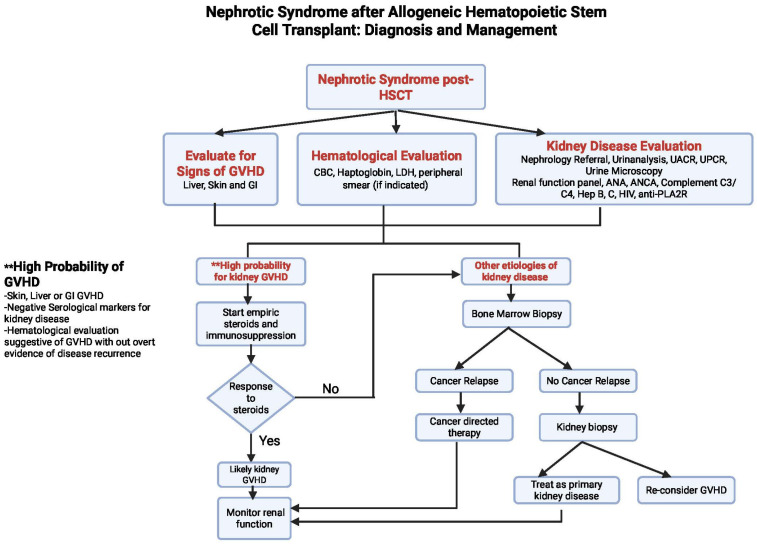
Proposed diagnostic and management algorithm for new onset nephrotic syndrome after allogeneic stem cell transplantation. Created in BioRender. Amarapurkar, P. (2026) https://BioRender.com/0z1iup5.

Management of paraneoplastic MCD in the setting of active AML after HSCT presents a distinct clinical challenge. Corticosteroids and immunosuppressive agents are standard first-line therapy for idiopathic MCD, but steroid resistance is well documented in malignancy-associated cases, as was observed in both of our patients. In the post-HSCT setting, intensifying immunosuppression carries the further risk of impairing the graft-versus-leukemia (GVL) effect. Recognizing the paraneoplastic etiology early avoids this hazard and redirects treatment toward the underlying malignancy. The available evidence consistently identifies effective control of the underlying neoplasm as the most critical determinant of renal recovery in paraneoplastic NS [18]. To support this approach in AML, we propose an algorithm for the diagnosis and management of paraneoplastic nephrotic syndrome associated with AML (Figure 2).

Time to renal remission varies by histologic subtype; prior series of post-HSCT NS have demonstrated faster recovery in MCD than in MN, with median remission times of approximately 1.8 versus 7 months, respectively [11]. Both of our cases are broadly consistent with this, though the pace of renal recovery appeared closely tied to leukemia response rather than to the histologic subtype alone. In Case 1, dialysis was discontinued following hematologic remission. In Case 2, proteinuria fell substantially within three days of the first leukemia treatment cycle, a time course that predates any meaningful immunosuppressive effect of prednisone on MCD.

## 4. Conclusions

These two cases expand the differential diagnosis of post-HSCT nephrotic syndrome beyond GVHD and immunosuppression withdrawal. They suggest that paraneoplastic MCD may, in some patients, represent an early manifestation of AML relapse after allogeneic HSCT, although further research is required for confirmation. When nephrotic syndrome develops in the post-HSCT setting without overt GVHD activity, or fails to respond to corticosteroids, evaluation for disease relapse warrants consideration. In such cases, recognizing a possible paraneoplastic contribution to the glomerular disease could inform treatment strategy, shifting therapeutic focus from intensification of immunosuppression toward cytoreduction, which may be essential to achieving renal recovery.

## Figures and Tables

**Table 1:** Patient labs at presentation.

Parameter	Case 1	Case 2	Reference Range
Serum creatinine (mg/dL)	3.14	1.8	0.5–1.4
BUN (mg/dL)	32	43	8–26
eGFR (mL/min/1.73 m^2^)	21.6	40	>60
Serum albumin (g/dL)	1.7	2.1	3.4–5.0
WBC (×10^9^/L)	3.5	2.4	3.8–10.6
ANC (×10^9^/L)	0.8	0.0	2.24–7.68
Circulating blasts (%)	0	41	0–0
Hemoglobin (g/dL)	14.1	9.7	12.9–16.9
Platelets (×10^9^/L)	123	25	156–369

**Table 2:** Reported cases of nephrotic syndrome associated with acute myeloid leukemia.

Author; Year [Ref]	Diagnosis (Dx)	Age, Sex	NS Timing vs. Dx/Relapse	Renal Biopsy Diagnosis	Urine Protein; Serum Creatinine	Leukemia Tx → Response	NS Therapy → Response	Suspected Etiology of NS
Dosa et al.; 1983 [22]	AML	57 M	Episode 1: 2 mo after dx	FSGS	2–3.3 g/day; UNK	VCR/pred → PR; Ara-C/dauno/TG/pred → CR	Pred → PR; CR with chemotherapy	AML
			Episode 2: few wk before relapse		3 g/day; 4.2 mg/dL	Chemo → NA (death)	Steroids + chemo → UNK	
Thomson et al.; 1989 [23]	AML	33 M	15 days after dx	MCD	22.7 g/day; UNK	Ara-C/dauno → NA (death)	Steroids + chemo → CR	AML vs. anthracycline
Morino et al.; 1995 [24]	APL	32 F	10 days after dx	Anthracycline nephropathy	17.5 g/day; UNK	BHAC-DMP → NA (death)	None	Anthracycline
Omura et al.; 1996 [25]	AML	59 M	26 wk after dx; AML in CR	GN (mesangial, crescentic), macrophage infiltration	25 g/day; 0.7 mg/dL	Multi-agent chemo + M-CSF → CR	Steroids + heparin + dipyridamole; stop M-CSF → PR	AML and M-CSF
Levi et al.; 2002 [26]	AML	44 F	2 mo before dx	Suspected MCD *	5.5 g/day; 0.8 mg/dL	Ara-C/mitoxantrone → CR	None; CR with chemo	AML
Sahiner et al.; 2004 [27]	AML	53 M	3–4 mo before dx	MN	4.4 g/day; 1.6 mg/dL	Ara-C/daunorubicin → UNK	Steroids + cyclophosphamide → PR; CR with chemo	AML
Singh et al.; 2004 [28]	AML	42 M	9 days after dx	Fibrillary GN	14 g/day; 0.7 mg/dL	Ara-C/dauno → CR	None; No renal response to chemo	AML
Stevenson et al.; 2005 [29]	AML; post HSCT	31 F	1.5 mo before relapse	MCD	3.9 g/day; UNK	None; died after AML relapse	Steroids + CSA → No response	AML
Stevenson et al.; 2005 [29]	AML; post HSCT	41 M	3.5 mo before relapse	MN	14.6 g/day; UNK	None; died after AML relapse	Steroids + CSA → No response	AML
Bariş et al.; 2010 [30]	APL	9 M	8 wk after dx; APL in CR	FSGS	6.2 g/day; 0.4 mg/dL	Ara-C/ida/etoposide/ATRA → CR	Steroids → CR	Anthracycline
Sethna et al.; 2012 [31]	APL	3 M	9 days after dx	Uncertain *	75 mg/dL †; 0.27 mg/dL	Ara-C/dauno/ATRA → CR	Steroids + stop ATRA → CR	Differentiation syndrome
Moradveisi et al.; 2014 [32]	APL	5 M	9 days after dx	Suspected MCD *	1 g/d; UNK	Ara-C/dauno/TG/ATRA → UNK	Steroids → CR	APL vs. anthracycline
Regino et al.; 2023 [33]	APL	28 M	1 wk before dx	GN *	4.6 g/day; normal	ATRA/ATO → CR	ATRA/ATO + ACEi/CCB → CR	APL

* Biopsy precluded; diagnosis inferred clinically. † Spot urine concentration (mg/dL); 24-h excretion not reported. Abbreviations: AML: acute myeloid leukemia; APL: acute promyelocytic leukemia; Ara-C: cytarabine arabinoside; ATRA: all-trans retinoic acid; ATO: arsenic trioxide; BHAC-DMP: behenoyl cytosine arabinoside, daunorubicin, 6-mercaptopurine; CCB: calcium channel blocker; CR: complete response; CSA: cyclosporine; dauno: daunorubicin; dx: diagnosis; FSGS: focal segmental glomerulosclerosis; GN: glomerulonephritis; ida: idarubicin; M-CSF: macrophage colony-stimulating factor; MCD: minimal change disease; MN: membranous nephropathy; mo: month(s); HSCT: hematopoietic stem cell transplant; NA: not assessable; pred: prednisone; PR: partial response; TG: thioguanine; Tx: therapy; UNK: unknown; VCR: vincristine; wk: week(s).

## Data Availability

The data presented in this study are available on request from the corresponding author due to patient privacy.

## Abbreviations

The following abbreviations are used in this manuscript:

ANA	Antinuclear antibody
ANCA	Antineutrophil cytoplasmic antibody
anti-dsDNA	Anti-double-stranded DNA antibody
anti-GBM	Anti-glomerular basement membrane antibody
anti-PLA2R	Anti-phospholipase A2 receptor antibody
anti-PR3	Anti-proteinase 3 antibody
ASXL1	Additional sex combs-like 1
BCOR	BCL6 corepressor
BCR-ABL1	Breakpoint cluster region-Abelson 1
BUN	Blood urea nitrogen
CD34	Cluster of differentiation 34
eGFR	Estimated glomerular filtration rate
EZH2	Enhancer of zeste homolog 2
FISH	Fluorescence in situ hybridization
GI	Gastrointestinal
HIV	Human immunodeficiency virus
HLA	Human leukocyte antigen
IDH2	Isocitrate dehydrogenase 2
MDS	Myelodysplastic syndrome
RBCs	Red blood cells
TNF-α	Tumor necrosis factor-alpha
WBCs	White blood cells
